# Systematic review of associations between concomitant rheumatoid arthritis and peripheral arterial disease, health-related quality of life and functional capacity

**DOI:** 10.1007/s00296-022-05245-7

**Published:** 2022-11-30

**Authors:** Tayser Zoubi, Hendry Gordon

**Affiliations:** 1 College of Medicine and Public Health, Flinders University, Bedford Park, Adelaide, SA 5042 Australia; 2 School of Health and Life Sciences, Glasgow Caledonian University, Cowcaddens Road, Glasgow, G4 0BA Scotland

**Keywords:** Arthritis, Rheumatism (MeSh), Peripheral arterial disease (MeSh), Quality of life (MeSh), Health-related quality of life

## Abstract

Patients with rheumatoid arthritis (RA) are at an increased risk of cardiovascular disease and vascular morbidity. The association between peripheral arterial disease (PAD) and RA has not been previously investigated within the scope of a review. Conjoined disease manifestations may impact patient well-being, perpetuating increased mortality and quality of life deficits. To investigate the association between RA and PAD, along with RA and the ankle-brachial pressure index (ABPI), the impact of disease concomitance on health-related quality of life (HRQOL) and functional capacity (FC) was also investigated. Individual study appraisal was completed using the Crowe Critical Appraisal Tool (CCAT). A level of evidence analysis was conducted using the American Society of Plastic Surgeons (ASPS) Evidence Rating Scale for Prognostic/Risk Studies. AMED^®^, CINAHL^®^, Health Source: Nursing/Academic Edition, MEDLINE^®^, AHFS^®^, Scopus, Web of Science, Cochrane Library and Google scholar. Ten studies produced a CCAT rating of ≥ 30 (75%) and were deemed high quality, while a single study demonstrated a score of 26 (65%) suggesting moderate quality. A grade “II” levels of evidence was awarded to positive association between RA and PAD. A gradation of “I” was awarded to the association between ABPI and RA. The impact of concomitant manifestations on HRQOL and FC did not qualify for a level of evidence analysis. The systematic inflammatory nature of RA likely contributes to the increased incidence of PAD within the population. Further investigations are required to ascertain the impact of conjoined disease manifestations on HRQOL and FC.

## Introduction

Rheumatoid arthritis (RA) is an autoimmune inflammatory disorder of the synovial tissue [1]. It is associated with increases in patient morbidity and mortality [2, 3]. RA affects 1% of the general population, with early intervention significantly mediating a favorable prognosis [4]. Ongoing medical innovations in biologic agents and disease-modifying drugs (DMARDs) have facilitated improved clinical outcomes [5]. In recent decades, RA has been denoted as an independent cardiovascular disease (CVD) risk factor [5]. This risk is perpetuated by the use of steroid-derived medication in the management of acute RA exacerbations, atherosclerotic changes inherent to the systemic inflammatory nature of RA, and the prevalence of vascular morbidities, including peripheral arterial disease (PAD) [2].

The underlying atherosclerotic mechanisms responsible for CVD within RA have also been suggested to impact the incidence of PAD [6]. Despite reported decreases in CVD risk secondary to the implementation of DMARDs and biologics, their impact on symptomatic or asymptomatic PAD development in the RA population is unknown [7, 8]. PAD is often associated with medium- and large-sized blood vessels of the lower limb, commonly the infra-inguinal or aortoiliac arteries [9]. Diagnosis of PAD is established through the ankle-brachial systolic pressure index (ABPI), with a score of ≤ 0.90 typically acting as the diagnostic threshold [10]. Secondary to PAD, patients may present with intermittent claudication (IC), manifesting as pain on movement of the lower limbs; or critical limb ischemia (ABPI ≤ 0.3) marked by rest pains, ulcerations and gangrene development [9]. In 2% of cases, amputation becomes a necessary clinical consideration [11]. Some patients describe the incidence of IC as an inconvenience, while others denote significant lifestyle detriments, social isolation and unemployment [11]. Management of symptoms thus becomes paramount in mitigating detriments to patient functional capacity (FC) and improving health-related quality of life (HRQOL).

As an independent disease entity, PAD correlates with premature mortality [12]. It also acts to perpetuate cardiovascular and cerebrovascular events at a two- to six-fold increased risk [12, 13]. The former remains valid irrespective of an asymptomatic or symptomatic disease manifestation [14]. McDermott et al. [15] concluded that individuals diagnosed with the asymptomatic variant of PAD present with poorer quality of life (QOL), FC and adverse calf muscle characteristics when compared to non-PAD controls. Treatment is carried out to reduce the risk of CVD events and manage lower extremity symptoms including activity limitations they impose [11]. The potential association between RA and PAD has not previously been formally investigated by means of a systematic review.

Makismovic et al. [16] suggested that patients with PAD demonstrate significantly lower physical functioning, social functioning and mental health when compared to healthy controls. The former deficits are magnified in patients with PAD presenting with IC or gangrene formation. Similarly, Tothova et al. [17] demonstrated a statistically significant difference between healthy individuals and patients with RA concerning physical health, level of independence, spirituality/religion/personal belief and the environmental domains pertinent to the WHOQOL-100 questionnaire. Considering FC alone, the literature correlates low ABPI scores negatively with 6-min walk times [18]. This functional decline is more pronounced in females [18]. Likewise, disease activity of patients with RA tends to negatively correlate with FC. Patients with RA in a state of remission as defined by ACR/EULAR criteria pose similar FC to healthy age–sex-matched controls, while patients presenting with elevated disease activity tend to demonstrate greater functional limitations and diminished HRQOL [19]. Despite independent RA and PAD manifestations’ impact on FC and HRQOL, reviews discussing their concomitant impact on the noted outcomes are largely absent.

RA and PAD manifestations may have a deleterious influence on a patient’s activity levels and concomitantly impact patient morbidity and mortality. Both RA and PAD are associated with suboptimal exercise levels, despite the established benefits exercise elicits to HRQOL, FC and complication risk [15, 20]. In states of elevated RA disease activity, severe disease manifestations, and multi-morbid presentations, symptomatic PAD progression may become accentuated. Moreover, symptomatic PAD progression may act to exacerbate the concomitant presentation’s impact on HRQOL and FC. In RA and PAD manifestations, IC presents a prevalence of 29% [15]. The incidence of symptomatic PAD in RA may cyclically act to further reduce activity levels, manifesting in further HRQOL and FC detriments. Understanding the association between the two disorders may facilitate early detection of PAD in patients with RA, enabling early intervention and prevention of subsequent complications. Currently, SIGN [21] and NICE [22] RA guidelines do not directly take into consideration the vascular detriments that RA poses. Exercise emphasis in these publications as it pertains to vascular health is largely absent.

This systematic review attempted to (1a) investigate the association between RA and PAD. As a subsect of the former, (1b) The association between RA and ABPI scores was examined. This review further aimed to (2) denote the significance of RA and PAD concomitance on HRQOL and FC.

## Methods

### Search strategy

The following electronic databases were examined: AMED^®^ (The Allied and Complementary Medicine Database), CINAHL^®^ (Cumulative Index of Nursing and Allied Health Literature), Health Source: Nursing/Academic Edition, Web of Science (WoS), Scopus, MEDLINE^®^, AHFS^®^—Consumer Medication Information and the Cochrane Library. Google Scholar was the only search engine employed to supplement the examined databases. These databases were considered appropriate due to their inclusion of literature pertinent to the biomedical sciences, arthritis and rheumatology, and the global allied health professional and medical disciplines. The search strategy comprised two searches implemented to address the primary and secondary aims of this review. The search strategy was completed between March 3, 2019 and October 1, 2022. In addition, reference lists of pertinent articles were investigated for relevant literature.

Publication dates were set at the maximum possible afforded by the relative databases. Table 1 provides an overview of the search terms, MeSh terms and Boolean operators relevant to the first and second literature searches conducted. Abbreviations and truncations of search terms were used as appropriate to ensure comprehensiveness.

**Table 1:** Keywords, MeSh terms and Boolean operators pertinent to the first review objective (First search conducted) and secondary objective (Second search conducted) in the EBSCO search of relevant databases

Second search conducted				
First search conducted				
OR		OR		OR
Inflammatory joint conditions	AND	Ankle brachial index	AND	Functional capacity
Arthritis, rheumatism (MeSh)		Ankle brachial pressure index		Health-related quality of life
Rheumatoid arthritis		Intermittent claudication		Disease burden
		Toe pressure brachial index		Poor health
		Limb ischemia		Quality of life (MeSh)
		Critical limb ischemia		
		Peripheral vascular diseases (MeSh)		
		Peripheral arterial disease (MeSh)		
		Peripheral arterial obstructive disease		
		Subclinical femoral atherosclerosis		

### Study selection

Cohort, case–control and cross-sectional studies were included. Randomized control trials (RCT) were considered as well if relevant prevalence or association data could be derived. Hospital audits and case reviews were omitted given their inability to garner sufficient data suggestive of a potential association [23]. Other inclusion criteria included the following:

- A clinically established RA diagnosis garnered by a rheumatologist using 1987 ACR or 2010 ACR/EULAR criteria.
- A clinically justified PAD diagnosis. PAD is typically considered with an ABPI ≤ 0.90 [9]. Diagnostic interpretations of ≤ 1.0 were still included if appropriate justification was provided. An ABPI of ≤ 1.0 is considered indicative of atherosclerotic changes and has been previously used as a PAD diagnostic threshold [24].
- Studies discussing an association between RA and PAD.
- Studies investigating the association between RA and ABPI independent of a PAD diagnosis.

**Fig. 1:**
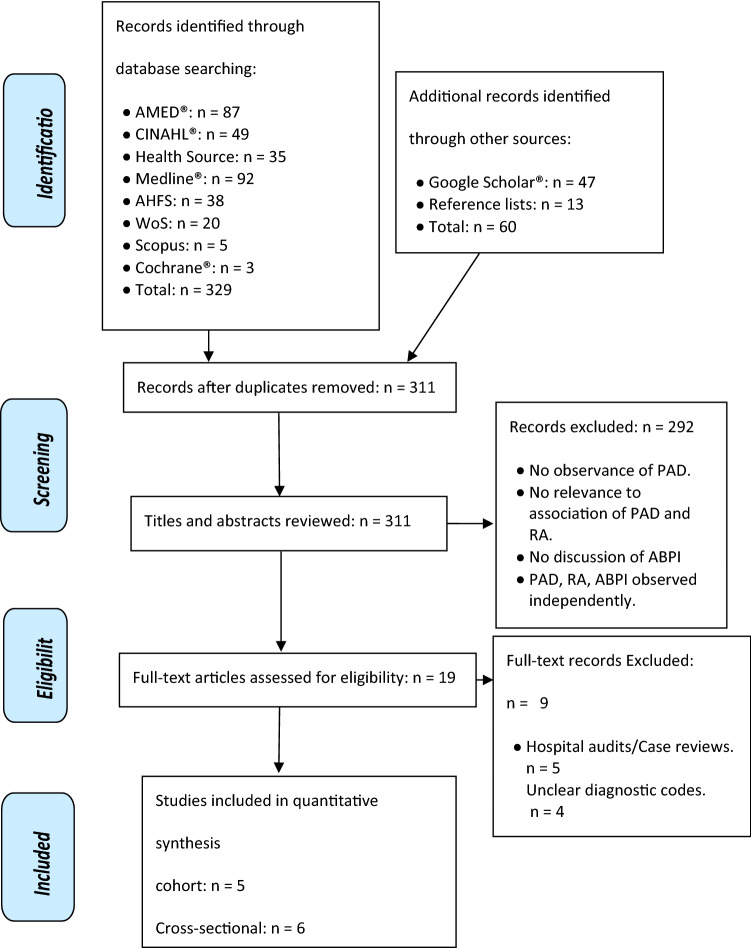
Result of search conducted between 3/3/2019 and 1/10/2022

Research investigating HRQOL, FC and the impact of IC on the former in conjoined disease manifestation is scarce. The noted outcome measures were not implemented as inclusion criteria due to this. The retrieval and filtering process is summarized in Fig. 1.

### Risk of bias assessment

The methodological rigor and quality of included studies were appraised using the Crowe Critical Appraisal Tool (CCAT) (Table 2) [24]. This tool was developed to encompass most study designs, including the ones pertinent to this review [25]. It consists of eight groupings: preliminaries, introduction, design, sampling, data collection, ethical matters, results and discussion [25]. Each grouping contains 22 items, carrying 98 item descriptors [25]. Groupings are individually scored between 0 and 5, determined by a piece of literature’s perceived adherence to the group items listed. A score of 0 denotes no evidence of item use, while integers 1, 2, 3, 4 and 5 suggest growing tiers of item use respectively (Table 2) [26]. A total score is then derived. A CCAT score of ≥ 30 (75%) denotes a high-quality design, a score between 22 (55%) and 26 (65%) suggests a moderate-quality design, and a score of ≤ 22 (55%) is deemed poor. This tool is accompanied by a comprehensive user guide in ensuring the attainment of valid scores when one conducts an appraisal [26]. Both the complete checklist and guide can be accessed through Crowe [27]. Crowe and Sheppard [27] demonstrated that the tool presents a good degree of construct validity when compared to the Physiotherapy Evidence Database (PEDro) scale, Cho and Bero scale, Single-Case Experimental Design (SCED) scale, and the Assessment of Multiple Systematic Reviews (AMSTAR) scale. A second study suggested the CCAT generates significant absolute agreement ICCs in the individual research designs tested [28]. Sufficient G scores were demonstrated by the CCAT as well, accentuating its reliability in appraising multiple study designs. Crow, Sheppard and Campbell [25] verified the CCAT’s capacity to diminish the influence of researcher experience on the validity and reliability of scoring.

**Table 2:** CCAT groupings and items [27]

CCAT groupings	Group items
Preliminaries	1. Title
	2. Abstract (assessed last)
	3. Text (assessed last)
Introduction	1. Background information
	2. Objective
Design	1. Research design
	2. Intervention, treatment, exposure
	3. Outcome, output, predictor, measure
	4. Bias, etc.
Sampling	1. Sampling method
	2. Sample size
	3. Sampling protocol
Data collection	1. Collection method
	2. Collection protocol
Ethical matters	1. Participant ethics
	2. Researcher ethics
Results	1. Analysis, integration, interpretation method
	2. Essential analysis
	3. Outcome, output, predictor analysis
Discussion	1. Interpretation
	2. Generalization
	3. Concluding remarks

## Results

Figure 1 outlines the strategy used in acquiring relevant studies. The search strategy resulted in 324 articles. 311 remained following duplicate removal. Following the process of title and abstract screening, 19 studies were eligible and included for full-text analysis. The most common reason for exclusion involved investigations of RA, PAD or ABPI independently, rather than conjoined manifestations or impact on relevant outcomes. A total of eleven studies met the inclusion criteria and were deemed appropriate for this review. Tables 3, 4 summarize the relevant literature found.

**Table 3:** Summary of included articles pertinent to the association between PAD and RA

Authors	Design	Sample size + mean age	Method of PAD diagnosis	Control inclusion	Confounders considered	Results	References
Chuang et al.	Retrospective cohort	RA Group: 23, 800 female 6969 male Mean age 53.7	National Health Insurance Research Database (NHIRD)	Random selection. Matched (1:1) according to age, sex and RA diagnosis index year	Age, sex and comorbidities (DM, hypertension, hyperlipidemia, COPD, heart failure, CAD and stroke)	Suggests association between PAD in RA	[36]
						6.27 per 1000 person-years (Adjusted HR 1.73, 95% CI 1.57–1.91)	
		Control: 23, 776 female 6,965 male Mean age 53.2				Six or more comorbidities (Adjusted HR-10.1, 95% CI 5.09–20.0)	
Bacani et al.	Retrospective Cohort	RA group: 326 female 144 male Mean age 55.6	Olmsted County, Minnesota medical records	Local residents of similar age, sex and indexed in accordance with RA incidence of corresponding patient	Cigarette smoking status, presence of dyslipidemia, hypertension or personal history of cardiovascular disease	Association between RA and PVEs including PAD	[34]
						Incidence 3.3 ± 1.1 versus 3.8 ± 1.4; *P* = 0.58	
		Control: 326 female 144 male Mean age 55.5				HR 1.5 [95% CI 0.6–3.7]	
						PAD—3.9 per 1000 person-years (95% CI 1.9–7.1)	
Kim et al.	Cross-sectional	RA group: 212 female 50 male Mean age 56.7	ABPI using VP-2000, Colin Co., Ltd., Komaki, Japan	Not applicable	Systolic blood pressure, age, diastolic blood pressure, triglycerides, total cholesterol, hemoglobin, swollen joint count, CRP and ESR	PAD prevalence 1.5% in RA Cohort	[30]
Liang et al.	Retrospective cohort	RA group: 445 female 164 male	Olmsted County, Minnesota medical records	Not applicable	Age, sex, smoking history at time of diagnosis, BMI, and RF at diagnosis	Extra-articular RA is causally linked to PAD	[35]
						HR 2.29, 95% CI 1.20–4.34	
Alkaabi et al.	Cross-sectional	RA group: 20 female 20 male Mean age 56	ABPI < 1.0 and Doppler ultrasound velocity detected	Matched for age, sex and postal code	High blood pressure, blood sugar, lipids, steroid usage, smoking status	Increased PAOD incidence in RA group	[24]
						ABPI < 1.0 (*P* = 0.007, Fisher’s test)	
		Control: 20 female 20 male Mean age 55				ABPI < 0.90 (*P* = 0.026, Fisher’s test)	
						ABPI < 1.0 demonstrated higher HAQ scores (*P* = 0.01, Mann Whitney *U* test)	

Authors	Design	Sample size + mean age	Method of PAD diagnosis	Control inclusion	Confounders considered	Results	References
Grech et al.	Cross-sectional	RA group: 84 female 16 male	The Huntleigh^®^ Dopplex Assist vascular package	Not applicable	CRP, ESR, RF, lipid profile, BMI, hypertension and smoking history	ABPI ≤ 0.90: found in 4% of subjects	[31]
						Doppler waveform analysis: 33.3% of subjects demonstrated impaired vascular function	
Turresson et al.	Retrospective cohort	RA Group: 185 Extra-articular RA group: 81	Medical records in accordance with an ABPI ≤ 0.90	Derived from medical database	Age, sex, smoking, RF factor seropositivity	Severe extra-articular RA Increases susceptibility to CVEs.s	[37]
						HR: 3.78; 95% CI 2.00–7.16	

*RA* rheumatoid arthritis, *PAD* peripheral arterial disease, *COPD* chronic obstructive pulmonary disease, *CAD* coronary artery disease, *CRP* C-reactive protein, *ESR* erythrocyte sedimentation rate, *RF* rheumatoid factor, *BMI* body mass index, *DM* diabetes mellitus, *CVE* cardiovascular event, *ABPI* ankle-brachial pressure index, *PVE* peripheral vascular events

**Table 4:** Summary of included articles pertinent to the association between ABPI and RA

Authors	Design	Sample size + mean age	Method of ABPI attainment	Control inclusion	Confounders considered	Results	References
Del-Rincon et al.	Prospective cohort	RA group: 210 female 24 male Mean age 59	Parks 8.1 MHz pocket Doppler probe	Self-reported good health, no history of smoking, no rheumatoid conditions and BMI < 30 kg/m^2	Cardiovascular risk factors, age, sex, diabetes, hypercholesterolemia, inflammatory markers and glucocorticoid dosage	Association between RA and reduced ABPI scores persists	[38]
		Control: 90 female 12 male Mean age 59				ABPI < 0.9 3% of RA group (*P* = 0.009)	
						Multinomial logistic regression suggestive of inflammatory marker involvement (OR 4.02; 95% CI 0.95–17.01)	
						Arthritis stratification against deformed joint count associated with obstruction (OR 7.16; 95% CI, 1.69–30.3)	
Kumeda et al.	Cross-sectional	RA group: 122 female 16 male Mean age 55	Mean bilateral ankles BP	Comparable in age, sex, blood pressure, serum lipid levels, menopause status, smoking status and BMI	Hypertension or use of associated medication, hyperlipidemia or associated medication, diabetes mellitus or associated medication, and history of cerebrovascular events of heart disease	ABPI was significantly lower in RA group	[29]
		Control: 85 female 9 male Mean age 52.1				*P* = 0.0206	
Fan et al.	Cross-sectional	RA group: 21 female 22 male Mean age 50	ABPI measured using automated oscillometric machine (Colin VP-1000 Plus)	Age- and sex-matched healthy volunteers	Fasting plasma lipids, high-density lipoprotein cholesterol, glucose levels and blood pressure	RA significant determinant of reduced ABPI	[32]
		Control: 34 female 39 male Mean age 51				ABPI lower in women (1.08 ± 0.06 vs. 1.11 ± 0.07, *P* = 0.003)	

*RA* rheumatoid arthritis, *PAD* peripheral arterial disease, *COPD* chronic obstructive pulmonary disease, *CAD* coronary artery disease, *CRP* C-reactive protein, *ESR* erythrocyte sedimentation rate, *RF* rheumatoid factor, *BMI* body mass index, *DM* diabetes mellitus, *CVE* cardiovascular event, *ABPI* ankle-brachial pressure index, *PVE* peripheral vascular events

Six studies were of a cross-sectional design [24, 29–33]. Four studies involved a cohort design, three of which were of a retrospective nature [34–37] and one implemented a prospective design [38]. Eight studies discussed the association between PAD and RA [24, 30, 31, 33–37] while the remaining three queried an association between ABPI and RA [29, 32, 38]. Three cross-sectional studies investigated concomitant disease impact on HRQOL and FC [24, 33, 39]. Outcome measures employed by relevant studies to ascertain HRQOL and FC involved the Modified Health Assessment Questionnaire (M-HAQ), the Health Assessment Questionnaire (HAQ) and the 6-m walk test [24, 29, 33]. None of the studies investigated the incidence of symptomatic IC in conjoined RA and PAD manifestations or discussed its role in perpetuating HRQOL and FC detriments.

Table 5 outlines the results of the CCAP study quality assessment. All studies pertinent to this review presented with a total % CCAP score of ≥ 60%. Bacani et al. [34], Chuang et al. [36], Grech et al. [31] and Turesson et al. [37] produced the highest totals, 90%, 93%, 90% and 92.5% respectively. The majority of studies produced a group item score of ≥ 3, adhering to most sub-item CCAT classifications [26]. Some articles neglected to discuss potential bias involved in their design [24, 29, 31, 32, 36]. The impact of potential selection bias, detection bias and categorical bias on result generalizability was discussed in five articles [30, 34, 35, 37, 38]. Discussion pertinent to conflict of interest and sources of funding was omitted by a single article [29].

**Table 5:** Quality Analysis derived from CCAP To

Study	Preliminaries	Introduction	Design	Sampling	Data collection	Ethical matters	Results	Discussion	Total/40	Total %	Quality descriptor	Reference
Fan et al.	5	5	3	3	5	4	4	5	34	85	High	[32]
Bacani et al.	5	5	5	3	5	4	4	5	36	90	High	[34]
Liang et al.	5	5	6	4	5	2	4	3	31	78	High	[35]
Kumeda et al.	5	5	3	3	4	1	5	4	30	75	High	[29]
Tehan et al.	5	5	4	3	4	5	4	4	34	85	High	[33]
Del Rincon et al.	5	5	5	3	4	3	4	4	31	75	High	[38]
Kim et al.	5	5	4	3	2	4	4	4	26	65	Moderate	[30]
Chuang et al.	5	5	4	4	5	5	4	5	37	93	High	[36]
Al Kaabi et al.	5	5	3	3	3	4	4	4	31	78	High	[24]
Turresson et al.	5	5	4	5	4	5	4	5	37	92.5	High	[37]
Grech et al.	5	5	5	4	4	5	4	4	36	90	High	[31]

Evidence pertaining to the positive association between RA and PAD produced a “II” level of evidence grading, due to the retrospective cohort studies found. The positive association between ABPI and RA was awarded a level of evidence rating of “I,” in adherence to the prospective single-catered design ASPS scale criterion. Lastly, the concomitant disease manifestation's effect on disease burden and poor health, defined through HRQOL and FC, did not qualify for a level of evidence classification due to the cross-sectional designs implemented [24, 29, 33].

### RA/PAD association

Five studies discussed the role of PAD in RA [24, 30, 31, 36, 37]. One of which was a retrospective cohort querying the incidence of PAD and its association with RA [36]. The authors demonstrated an overall incidence of 6.27 per 1000 person-years, posing an adjusted hazard ratio (HR) of 1.73 (95% CI 1.21–1.83) [36]. Disease development risk peaked during the first follow-up year (adjusted HR 2.42, 95% CI 1.92–3.05), and declined during the 4–5-year follow-up period (adjusted HR 1.45, 95% CI 1.17–1.81) [36]. The sex-specific PAD development risk was higher in women (adjusted HR 1.80, 85% CI 1.61–2.02) than in men (adjusted HR 1.49, 95% CI 1.21–1.83) [36]. Moreover, the relative increase in the number of comorbidities a patient presented with, resulted in higher HR values [36]. Relevant comorbidities included diabetes mellitus (DM), hypertension, hyperlipidemia, heart failure, coronary artery disease (CAD) and stroke [36]. Three studies of a cross-sectional nature were in agreement with an increased PAD prevalence in RA, while one was not [33]. The study in disagreement recruited participants in RA remission, demonstrating no differences in vascular outcomes compared to controls [33]. Al-Kaabi et al. [24] reported a 25% prevalence of PAD in patients with RA and a 2.5% prevalence among healthy controls (*P* = 0.007), in the unadjusted model used. PAD was defined as an ABPI ≤ 1.0. The systolic blood pressure was significantly higher in patients with RA when compared to healthy controls (mean 137 vs. 126 mmHg; *P* = 0.01). Grech et al. [31] noted a 4% prevalence of PAD, derived from ABPI, in the RA population examined. Vascular obstruction indicative of PAD was noted in 33.3% of the population when investigated using Doppler Spectral Waveform Sonography. No significant differences between groups were found regarding smoking [23, 31], diabetes mellitus [24], blood glucose [24, 31], serum cholesterol [24] and serum triglycerides [24, 31]. Kim et al. [30] denoted a PAD prevalence of 1.5% in the RA population studied.

### RA/ABPI association

Two of the studies reported a notable association between ABPI and RA [29, 38]. A single study presented RA as an independent risk factor for reduced ABPI scores but rejected the role inflammatory markers play in facilitating the latter [32]. Del-Rincon et al. [38] demonstrated that patients with RA presented with an adjusted odds ratio (OR) of 3.33 (95% CI 0.79–13.96, *P* = 0.02) in developing arterial obstruction (ABPI ≤ 0.90) when compared to healthy controls. The authors stratified for age, sex, diabetes mellitus (DM), hypercholesterolemia, systolic blood pressure and BMI. In considering the former confounding variables on arterial incompressibility (ABPI ≥ 1.3), an OR of 9.50 (2.03–44.5, *P* = 0.004) was found. The highest degree of obstruction was demonstrated among patients with RA carrying the greatest joint deformity tertials (≥ 20), producing a Relative-Risk Ratio (RR) of 7.6 (95% CI 1.02–1.06, *P* < 0.0001). Fan et al. [32] demonstrated no correlation between ABPI and plasma markers, inflammatory markers or endothelial markers among patients with RA. Investigated markers included CRP, Interleukin (IL)—1β, IL-6, tumor necrosis factor (TNF)–α, macrophage migration inhibitory factor (MIF) and von Willebrand factor (vWF). Kumeda et al. [29] reported a significantly lower ABPI in patients with RA when compared to healthy controls (*P* = 0.0206). No significant difference was noted with respect to age, sex, BMI, smoking index, serum lipid levels and systolic/diastolic BP between groups.

### Disease burden and poor health

Three cross-sectional studies considered the impact of concomitant PAD and RA on HRQOL and FC [24, 33, 39]. Patients with RA diagnosed with PAD elicited higher HAQ scores compared to patients with normal ABPI measures; mean S.E 1.7 (0.2) versus 0.78 (0.14), *P* = 0.01 respectively [24]. Al-Kaabi et al. [24] noted an IC prevalence of 7.5% in the conjoined RA and PAD population studied. The authors did not ascertain HRQOL and FC detriments imposed by IC in RA. No association was found in the RA group between inflammatory markers, disease duration or HAQ scores. Kumeda et al. [29] demonstrated reduced M-HAQ scores in the RA group when compared to healthy controls. This reduction best correlated with carotid intima-media thickness (0.305, *P* ≤ 0.05). No comparison between M-HAQ scores and ABPI ensued. These findings were independent of age, sex, smoking index, blood pressure, total cholesterol and blood triglyceride count of the examined populations. Tehan et al. [33] denoted reduced gait speed as a measure of FC in the RA populations with reduced ABPI scores. This correlation was not significant likely due to limited RA disease activity in the population studied.

## Discussion

This review considers the association between RA and PAD, coupled with their concomitant impact on HRQOL and FC. Four retrospective cohort studies investigating the association between RA and PAD were of a high-quality design [24, 34, 35, 37]. Moreover, four articles were cross-sectional, three of which demonstrated a high-quality design [24, 29, 31, 33], while a single article was of moderate quality [30]. Investigated articles isolating PAD in their analysis were in agreement with an increased PAD incidence or prevalence among patients with RA [24, 35–37]. One exception to the former persisted denoting no increase in prevalence between the RA and controls [33]. Grech et al. [31] questioned the validity of ABPI as a PAD diagnostic method in RA, suggesting that PAD remains substantially underdiagnosed. The examined literature determined that PAD incidence is associated with either extra-articular RA disease manifestations, the prevalence of comorbidities, or RA disease severity marked by CRP or ESR concentrations and joint deformity counts [35–38]. These findings suggested that disease manifestations of greater severity and chronicity would increase an RA patient’s susceptibility to PAD development. CVD risk factors at the time of RA diagnosis, including smoking history, are significantly associated with PAD development but are not independently responsible for its incidence [35]. The former ideation was echoed by other meta-analyses and systematic reviews concerning the association between RA and vascular morbidities [40–42]. These included venous thromboembolism (VTEs) [41] and CAD [40–42].

A grade “I” level of evidence was demonstrated concerning the positive association between ABPI and RA, suggesting RA as an independent risk factor for arterial obstruction or incompressibility [38]. One high-quality study presented the necessary “prospective cohort” and “single-centered” criterion proposed by the ASPS to award the noted gradation [38]. Two studies of a high-quality cross-sectional design noted an increased prevalence of reduced ABPI in patients with RA [29, 32]. This remained true when individuals diagnosed with PAD were excluded [32]. ABPI acts as a valid marker of generalized atherosclerotic changes concerning the peripheral [10, 21], carotid [41] and coronary [10] arterial beds. The association noted in this review may further validate ABPI as a marker of atherosclerosis in RA.

Articles addressing the impact of conjoint RA and PAD manifestations on HRQOL and FC measures were not eligible for a level of evidence gradation, despite a robust high-quality design [24, 29, 33]. Evidence appraised suggested diminished HRQOL and FC in concomitant disease manifestations [24]. This finding is independent of the reduced FC demonstrated by RA alone [39]. Detriments to HRQOL and FC more accurately correlated with RA severity and joint deformity, rather than the prevalence of PAD in concomitant manifestations [24]. The concomitance of RA and reduced ABPI was correlated with reduced FC in an RA population with non-significant disease activity [33]. No discussion ensued of the potential impact of symptomatic PAD on FC, HRQOL or perceived disease burden, despite denoting a 7.5% prevalence of IC among the RA population examined in one study [24]. Symptomatic PAD may reduce a patient’s HRQOL and FC as it often presents with pain on movement. Previously symptomatic patients with PAD demonstrated greater detriments to their HRQOL and FC when compared to non-symptomatic PAD counterparts, and were at risk of disease progression-related mobility loss [18, 43]. Investigating the impact of symptomatic PAD in RA remains paramount in improving patients’ prognostic outlook.

Individual study appraisal scores were determined using the CCAT, with all included studies demonstrating a robust design, carrying minimal potential for bias. Some studies, however, were subject to selection bias, limiting validity [30, 32]. Kim et al. [30] and Tehan et al. [33] for instance recruited RA patients with mild disease severity. Previous studies have denoted RA disease severity as a propagator of atherosclerotic changes[24, 31, 32, 34–37], suggesting the findings of Kim et al. [30] and Tehan et al. [33] lack generalizability. Fan et al. [32] recruited healthy controls from the risk evaluation clinic of Baker IDI Heart and Diabetes Institute, rather than from the community. This may have reduced the significance of their findings given that data derived from the control group may be subject to Berkson’s Bias [44]. Other studies derived their samples from a predominately ethnically homogenous population, reducing the potential implication of their findings on ethnically diverse groups [29, 34, 36]. Despite a degree of methodological homogeneity, evident heterogeneity was present regarding ABPI acquisition. Notably, studies either implemented manual Doppler [24, 30, 33, 38], sphygmomanometric methods [29], or automated oscillometry [30, 31] in deriving ABPI scores. Manual methodological approaches in ascertaining ABPI posit a degree of observer bias [45]. ABPI score inaccuracies may have been present since the observer must measure ankle and brachial pressures simultaneously rather than successively [45]. Despite reducing the risk of observer bias, no evidence was found validating automated oscillometry in an RA population.

RA has been previously documented as a novel CVD risk factor, with disease-associated cardiovascular event incidence similar to coronary heart disease (CHD) [46]. The presence of other CVD risk factors in RA has been demonstrated to accentuate vascular event risk [46]. The former is validated by the studies included in this review, where-by inclusion of CVD risk factors in individual studies perpetuated PAD incidence risk [24, 34–36]. All pertinent studies controlled for most noted CVD risk factors through exclusion or adjustment. However, 90% of reviewed studies neglected to control for physical activity levels, further limiting their respective internal validity [24, 30–32, 34–38]. The evidence suggests that patients with RA tend to exhibit more sedentary behavior when compared with population-based controls [47]. Despite this being likely due to pain, joint deformity and the prevalence of comorbidities, it may independently act to exaggerate PAD incidence within the population, affecting the internal validity and generalizability of appraised studies. This view is validated by Kumeda et al. [29], denoting that a lack of physical activity in the population perpetuates the multi-factorial incidence of arterial wall thickening, propagating atherosclerotic change.

This review has some limitations that warrant consideration. Available Journals were restricted to free-access organizations and those provided by Glasgow Caledonian University (GCU) access. This may have diminished the search strategy’s validity. Included studies consisted of peer-reviewed literature written in English exclusively, which may have acted to omit pertinent non-English literature. The appraisal and levels of evidence processes were conducted by a single researcher. CCAT and ASPS results may have been either attenuated or accentuated due to human error, limiting the validity of the results.

The implementation of exercise in the management of RA has been demonstrated to increase patient self-efficacy and reduce joint morning stiffness [21]. This benefit was best enabled when mild to moderate aerobic exercise was implemented [21]. The noted exercise intensity has been shown to combat disease-related muscle wasting and diminished fitness levels, which may improve HRQOL and act to attenuate morbidity [21]. Considering PAD, exercise therapy acts as a gold-standard first-line approach to treatment [22]. It is paramount in alleviating the risk of symptomatic disease or reducing the impact of symptomatic manifestations [22]. Exercise may be prominent in combating the HRQOL and FC hindrances that are likely present in conjoined RA and symptomatic/asymptomatic PAD manifestations [48]. Individualized exercise regimes have demonstrated improvements in endothelial function among patients with RA [49].

Future research would benefit from stratifying for physical activity levels and investigating the impact of symptomatic PAD on the relevant outcomes. Studies appraised did not implement exercise as a risk factor due to logistic difficulty, the subjectivity of self-reported measures and the inadequacy of medical databases used. No cohort or case–control studies produced by the search strategy implemented discussed the impact of conjoined RA and PAD on HRQOL and FC. Undertaking prospective cohort studies to improve the level of evidence pertaining to the subject is necessary for perpetuating the appropriate guideline revisions.

To conclude, the evidence presented in this review demonstrated an association between RA and PAD. Multi-morbid patient presentations act to accentuate the incidence risk of PAD in RA. Little evidence was found articulating the impact of conjoined disease manifestations on HRQOL and FC. Future studies will benefit from controlling for exercise as a prominent PAD risk factor and establishing the impact of symptomatic PAD on HRQOL and FC in the RA population.
